# Effectiveness of extracellular vesicles derived from hiPSCs in repairing hyperoxia-induced injury in a fetal murine lung explant model

**DOI:** 10.1186/s13287-024-03687-3

**Published:** 2024-03-14

**Authors:** Hala Saneh, Heather Wanczyk, Joanne Walker, Christine Finck

**Affiliations:** 1 Department of Neonatal Medicine, Connecticut Children’s Medical Center, Hartford, CT USA; 2 Department of Pediatrics, University of Connecticut Health Center, Farmington, CT USA; 3 Department of Pediatric Surgery, Connecticut Children’s Medical Center, Hartford, CT USA

**Keywords:** Bronchopulmonary dysplasia, Prematurity, Lung injury, Human induced pluripotent stem cells, Extracellular vesicles

## Abstract

**Background:**

Despite advances in neonatal care, the incidence of Bronchopulmonary Dysplasia (BPD) remains high among preterm infants. Human induced pluripotent stem cells (hiPSCs) have shown promise in repairing injury in animal BPD models. Evidence suggests they exert their effects via paracrine mechanisms. We aim herein to assess the effectiveness of extracellular vesicles (EVs) derived from hiPSCs and their alveolar progenies (diPSCs) in attenuating hyperoxic injury in a preterm lung explant model.

**Methods:**

Murine lung lobes were harvested on embryonic day 17.5 and maintained in air–liquid interface. Following exposure to 95% O_2_ for 24 h, media was supplemented with 5 × 10^6^ particles/mL of EVs isolated from hiPSCs or diPSCs by size-exclusion chromatography. On day 3, explants were assessed using Hematoxylin–Eosin staining with mean linear intercept (MLI) measurements, immunohistochemistry, *VEGFa* and antioxidant gene expression. Statistical analysis was conducted using one-way ANOVA and Multiple Comparison Test. EV proteomic profiling was performed, and annotations focused on alveolarization and angiogenesis signaling pathways, as well as anti-inflammatory, anti-oxidant, and regenerative pathways.

**Results:**

Exposure of fetal lung explants to hyperoxia induced airspace enlargement, increased MLI, upregulation of anti-oxidants *Prdx5* and *Nfe2l2* with decreased *VEGFa* expression. Treatment with hiPSC-EVs improved parenchymal histologic changes. No overt changes in vasculature structure were observed on immunohistochemistry in our in vitro model. However, *VEGFa* and anti-oxidant genes were upregulated with diPSC-EVs, suggesting a pro-angiogenic and cytoprotective potential. EV proteomic analysis provided new insights in regard to potential pathways influencing lung regeneration.

**Conclusion:**

This proof-of-concept in vitro study reveals a potential role for hiPSC- and diPSC-EVs in attenuating lung changes associated with prematurity and oxygen exposure. Our findings pave the way for a novel cell free approach to prevent and/or treat BPD, and ultimately reduce the global burden of the disease.

**Supplementary Information:**

The online version contains supplementary material available at 10.1186/s13287-024-03687-3.

## Background

Bronchopulmonary Dysplasia, or BPD, is a developmental lung disease characterized by a disruption or arrest of the normal processes of lung alveolarization and angiogenesis. Extreme preterm infants, born before 28 weeks’ gestation during the late canalicular or early saccular stages of their lung development, are mostly affected [1–3]. Gentle ventilation strategies, fluid restriction, caffeine, diuretics, and steroids, have all been used to prevent or attenuate lung injury; however, the incidence of BPD remains greater than 40% among extreme preterm infants [4], and there is still no effective therapy to restore normal alveolarization and angiogenesis.

Various types of stem cells, including mesenchymal stem cells (MSCs) and human induced pluripotent stem cells (hiPSCs), have shown promise in reducing inflammatory changes and restoring lung morphogenesis in experimental animal BPD models [5–9]. However, their potential immunogenicity remains a major limitation to their clinical applicability [10–12]. Tumorigenicity is also a concern, mostly with hiPSC-based treatments [13, 14]. The differentiation of hiPSCs into distal lung phenotypes (diPSCs, or differentiated hiPSCs) before their administration mitigates this risk [15, 16]; it might however reduce their effectiveness [5].

Recent evidence suggests that extracellular vesicles (EVs) secreted by stem cells play a significant role in mediating their therapeutic effects [7, 17–21]. EVs are nanoparticles secreted by most cells. They are capable of transferring biological components such as proteins, growth factors, RNAs and miRNAs to host cells or tissues, thus playing an important role in cellular communication and cell signaling. EVs have been shown to contribute to vital physiological functions, including homeostasis, immune regulation, and tissue regeneration and repair. In several diseases processes, including lung disease of prematurity, it has been shown that stem cell-conditioned media is as effective as the stem cells themselves in promoting tissue regeneration and repair. Furthermore, EVs are less likely to be immunogenic and tumorigenic than their parental cells. If effective, EV-based acellular approaches might then be safer than whole cells and may ease the transition of regenerative therapies towards clinical translation in BPD studies.

In this study, *we aimed to* evaluate the effectiveness of EVs derived from hiPSCs and diPSCs in attenuating hyperoxic injury in a fetal murine lung explant model. Our model is designed to recapitulate the clinical setting of extreme preterm birth and high oxygen exposure, which both result in the disruption of normal lung development.

## Methods

### hiPSC culture and differentiation

hiPSCs (passage 9) procured from ATCC (*American Type Culture Collection*) were plated at 2 × 10^4^ cells/cm^2^ in 6-well plates coated with 2 mg/mL Matrigel Matrix (*Corning*). hiPSC culture and differentiation were carried at 37 °C, under atmospheric oxygen tension (21% O_2_) and in 5% CO_2_. 10 ng/mL Rock Inhibitor Y27632 (*Tocris Bioscience*) was initially added to the medium (*NutriStem*) for 24 h to improve survival. Cells without Rock Inhibitor were then cultured for an additional 2–3 days until 70–80% confluency was reached. hiPSCs were passaged using ReLeSR (*StemCell Technologies*), seeded on 12-well Matrigel-coated plates, and differentiated towards a distal lung phenotype (diPSCs), following our lab’s previously published protocol [22]. Briefly, hiPSCs were exposed to growth factors to induce Definitive Endoderm (DE), Anterior Foregut Endoderm (AFE) and Lung Progenitor (LP) differentiation over 25 days. Following AFE induction phase, cells were split using TrypLE (*ThermoFisher*) to remove uncommitted cells and improve purity as previously described [22]. Cell characterization during the differentiation process is shown in Additional file 1: Fig. 1.

### Collection of cell-conditioned media and EV isolation

hiPSC-conditioned media was collected from cells after 48 h of culture. diPSC-conditioned media was collected following the 25-days differentiation process outlined above; at that time, cells were maintained in Dulbecco's Modified Eagle Medium (DMEM, *ThermoFisher*) supplemented with human serum replacement 50x (*Millipore Sigma*), penicillin/streptomycin solution 1×, Glutamax 1×, Non-essential Amino Acids 1× (*Gibco, ThermoFisher*), recombinant human fibroblast growth factors FGF2, FGF7, FGF10, and bone morphogenetic protein BMP4 (all growth factors from *Peprotech,* used at a final concentration of 10 ng/mL).

EVs were isolated from hiPSC- and diPSC-conditioned media by ultrafiltration (UF) combined with size-exclusion chromatography (SEC). Starting with 12 mL of cell culture supernatant, each sample was centrifuged sequentially at 500×*g* for 10 min to remove cellular debris, 10,000×*g* for 10 min to remove large microvesicles, then 3260×*g* for 12 min using Amicon Ultra-15 Centrifugal Filter Unit with a 50-kDa molecular weight cutoff (*Millipore Sigma*). In the last step, EVs were recovered in a total volume of 500 µL. Once the chromatography column (qEV original 35 nm, *IZON*) was equilibrated to room temperature and flushed with phosphate buffered saline (PBS), the concentrated EV sample was loaded, and the run-through collected. The buffer volume was first discarded, then three purified EV fractions (500 µL each) were collected. Fractions were characterized as follows below, then stored in PBS at − 20 °C. The second and third fractions (F2 and F3) were found to have the highest EV yield and were subsequently used in the lung explant experiments.

### EV characterization

The isolated EVs were characterized using four modalities: transmission electron microscopy (TEM), imaging flow cytometry (IFC), nanoparticle tracking analysis (NTA), and mass spectrometry (MS).

#### Transmission electron microscopy (TEM)

Carbon-coated copper grids were used for EV adsorption. Glow discharge was performed using a *Denton Vacuum 502B* to enhance adhesion, then 5 µL of EVs were deposited on each grid and allowed to adsorb for 2 min. After three brief washes with distilled water, grids were negatively stained with two droplets of 1% Uranyl Acetate and air-dried. Imaging was carried out using a *Hitachi H-7650* transmission electron microscope at 80 kV and *AMT Image Capture Engine*.

#### Imaging flow cytometry (IFC)

EV samples were stained with the following labels and monoclonal antibodies: CFDA-SE (carboxyfluorescein diacetate succinimidyl ester, *ThermoFisher*) at 2 µM, antiCD81-FITC (*ThermoFisher*) at 0.75 µg/mL (1:40 dilution), and antiCD63-PE (*ThermoFisher*) at 0.31 µg/mL (1:80 dilution).

CFDA-SE is a pan-EV label used to assess EV integrity. When CFDA-SE crosses intact membranes, the acetate group is cleaved by intra-vesicular esterases, resulting in the highly fluorescent CFSE molecule. CD81 and CD63 are tetraspanins present on the surface of some, but not all, EVs [23].

Monoclonal antibodies were first centrifuged at 17,000×*g* for 30 min (using centrifugal filter units with a 0.22-µm pore size, *Millipore*), to eliminate clumps or aggregates [24]. Antibody titrations were then performed using a series of dilutions in PBS (1:5, 1:10, 1:20, 1:40, 1:80, 1:160) to identify the optimal concentration that results in the highest signal-to-noise ratio. Fluorescent labels were added to 7.5 µL of EVs at the concentrations mentioned above to achieve a final volume of 30 µL per sample. Negative controls of buffers and antibodies (PBS alone, PBS with CFDA-SE, PBS with antiCD81-FITC, PBS with antiCD63-PE) were run to ensure the purity of PBS and the absence of antibody clumps. Single-stained samples were used to create compensation matrices. Double-staining of EVs with CFDA-SE/antiCD63-PE and antiCD81-FITC/antiCD63-PE were performed. Since significant spectral overlap exists between CFSE and FITC, co-staining with these labels was not done.

Multispectral imaging acquisition was performed using *Amnis ImageStreamX MKII (EMD Millipore)* [25, 26]. Calibration beads were run with each sample to verify optimal instrument performance. Fluidics were set to low speed, sensitivity to high, and magnification to 60×. High gain mode was turned on. Controls and EV samples were acquired over one minute each. Compensation matrices were created in the absence of brightfield illumination and side-scatter. Data analysis was performed using Image Data Exploration and Analysis Software (*IDEAS*).

#### Nanoparticle tracking analysis (NTA)

EV concentration and size distribution were measured by our collaborators at the University of Vermont (Weiss Lab) using a ZetaView NTA (*Particle Metrix*). All samples were diluted in Nanopure water to a final volume of 1 mL. 100-nm uniform beads were run as a positive control and for quality check before loading the samples. PBS run through the chromatography column was also tested for the presence of contaminants from the column. The captured videos were analyzed using ZetaView software.

#### Protein quantification and mass spectrometry (MS)

EV samples were lysed using radioimmunoprecipitation assay (RIPA) buffer and sonication. BCA assay (bicinchoninic acid assay kit, *ThermoFisher*) was performed to quantify proteins in the samples. Lysed EVs were then incubated overnight in 33% TCA (trichloroacetic acid) for protein denaturation. The next day, samples were centrifuged at 20,000×*g* for 20 min. The pellet was re-suspended in 500 µL of Acetone, and centrifuged again at 20,000×*g* for 20 min. The precipitated proteins were air-dried for 5 min then re-suspended in 1 M urea for mass spectrometry analysis.

Liquid chromatography–Mass Spectrometry (LCMS) was performed at the University of Connecticut proteomic core facility. Submitted samples were reduced with 5 mM dithiothreitol at room temperature, and alkylated with 10 mM iodoacetamide at room temperature, protected from light. Modified porcine trypsin protease (*Promega #V5113*) was added at a ratio of 1:20 w/w enzyme: protein at 37 °C and incubated overnight, and the digestion was quenched with formic acid. Peptides were then desalted by reverse-phase chromatography using Pierce peptide desalting spin columns (*Thermo Fisher part #89870*), dried completely, and resuspended in Solvent A (0.1% formic acid in Fisher Optima LC/MS grade water). Samples were quantified by A_280_ absorbance and total injection amount was normalized to 500 ng across samples.

Peptides were subjected to mass analysis using a Thermo Scientific Ultimate 3000 RSLC nano ultra-high performance liquid chromatography (UPLC) system coupled to a high-resolution Thermo Scientific Q-Exactive HF mass spectrometer. Each sample was injected onto a 25 cm C18 column held at 50 °C and separated by reversed-phase UPLC using a gradient of 4–90% Solvent B (0.1% formic acid in Fisher Optima LC/MS grade acetonitrile) over a 60-min gradient at 300 nL/min flow, followed by a 10-min wash and 20-min column re-equilibration. Peptides were eluted directly into the QE-HF using positive mode nanoflow electrospray ionization. MS1 scan parameters were set to 60,000 resolution, 1e^6^ AGC target, maximum ion time of 60 ms, and a mass range of 300–1800 m/*z*. MS2 data were acquired in data-dependent Top15 mode using the following parameters: 15,000 resolution, maximum ion time of 40 ms, isolation window of 2.0 m/*z*, 30 s dynamic exclusion window, normalized collision energy of 27, a scan range of 200–2000 m/*z*, and charge exclusion “on” for all unassigned, +1, and > + 8 charged species.

Peptides were identified using MaxQuant (v1.6.10.43) and its embedded Andromeda search engine and quantified by label-free quantification [27]. The raw data were searched against both the complete UniProt *Homo sapiens* reference proteome (identifier UP000005640, accessed 11Jan2022) and the MaxQuant contaminants database. Variable modifications allowed oxidation of Met, acetylation of protein N-termini, deamidation of Asn/Gln, and peptide N-terminal Gln to pyroGlu conversion. Carbamidomethylation of Cys was set as a fixed modification. Protease specificity was set to trypsin/P with a maximum of 2 missed cleavages. All results were filtered to a 1% false discovery rate at the peptide and protein levels using the target-decoy approach; all other parameters were kept at default values. MaxQuant output files were imported into Scaffold (*Proteome Software, Inc.*) and IPA (*Ingenuity Pathway Analysis, Qiagen*) for data visualization and subsequent analyses.

### Fetal lung tissue explant model

All animal experiments were carried out in accordance with protocols approved by the Institutional Animal Care and Use Committee (IACUC) at the University of Connecticut Health Center (Protocol number AP-200408-0224). C57/BL6 mice were bred and monitored for the presence of a plug. The day of vaginal plug visualization was designated as E0.5 (embryonic day 0.5). Dams were euthanized at E17.5 using CO_2_ narcosis, and laparotomy was performed to extract the fetuses. E17.5 corresponds to 24–26 weeks of human gestation, where lungs are at the late canalicular or early saccular stage of their development [10, 28–30]. Given the relative resistance of fetuses to the effects of CO_2_, cervical dislocation was also performed on fetuses following their extraction to ensure death prior to thoracotomy and lung tissue collection. Dams and fetuses’ death was confirmed by the cessation of respiration and loss of reflexes.

Fetal lung lobes were collected and washed in PBS and Hanks Balanced Salt Solution (HBSS). Ex vivo culture was carried at an air–liquid interface using an adapted protocol for culture of murine embryonic lungs [31]. Briefly, lung explants were placed over a trans-membrane insert and embedded in 15 µL of 8 mg/mL Matrigel solution (*Corning*). Plates were incubated at 37 °C for 2 h to allow for Matrigel gelation. DMEM:F12 medium (80:20) supplemented with 10% FBS (fetal bovine serum), penicillin/streptomycin solution 1×, Glutamax 1×, FGF2 at 25 ng/mL, FGF7 at 10 ng/mL and FGF10 at 25 ng/mL, was then added below the inserts. These growth factors concentrations were previously shown to promote alveolarization and angiogenesis in in vitro fetal lung models [32–35]. Culture in Matrigel at an air–liquid interface at 37 °C, as described, preserved the viability of the explant model for up to 7 days, as shown with Calcein-Ethidium tissue staining (Additional file 1: Fig. 2).

### Experimental design

#### Exposure to hyperoxia

After 4 h of in vitro culture in Matrigel under atmospheric oxygen tension (21% O_2_, 5% CO_2_) at 37 °C, plates were transferred into a modular incubator chamber MIC-101 (*Billups-Rothenberg*). Two petri dishes filled with sterile water were also placed inside the chamber to allow for humification. The chamber was then sealed and purged with a gas mixture of 95% O_2_/5% CO_2_ at 7 L/min for 13 min at 2 psi. Flushing this volume allows for a complete gas exchange. Once the purge was completed, the inlet port was disconnected from the gas source, and both inlet and outlet ports were clamped. The chamber was then placed back at 37 °C. The MIC-101 is designed to maintain constant gas levels for a minimum of 72 h. Explant models were exposed to 24 h of 95% O_2_ to induce airspace disruption and enlargement.

**Fig. 1:**
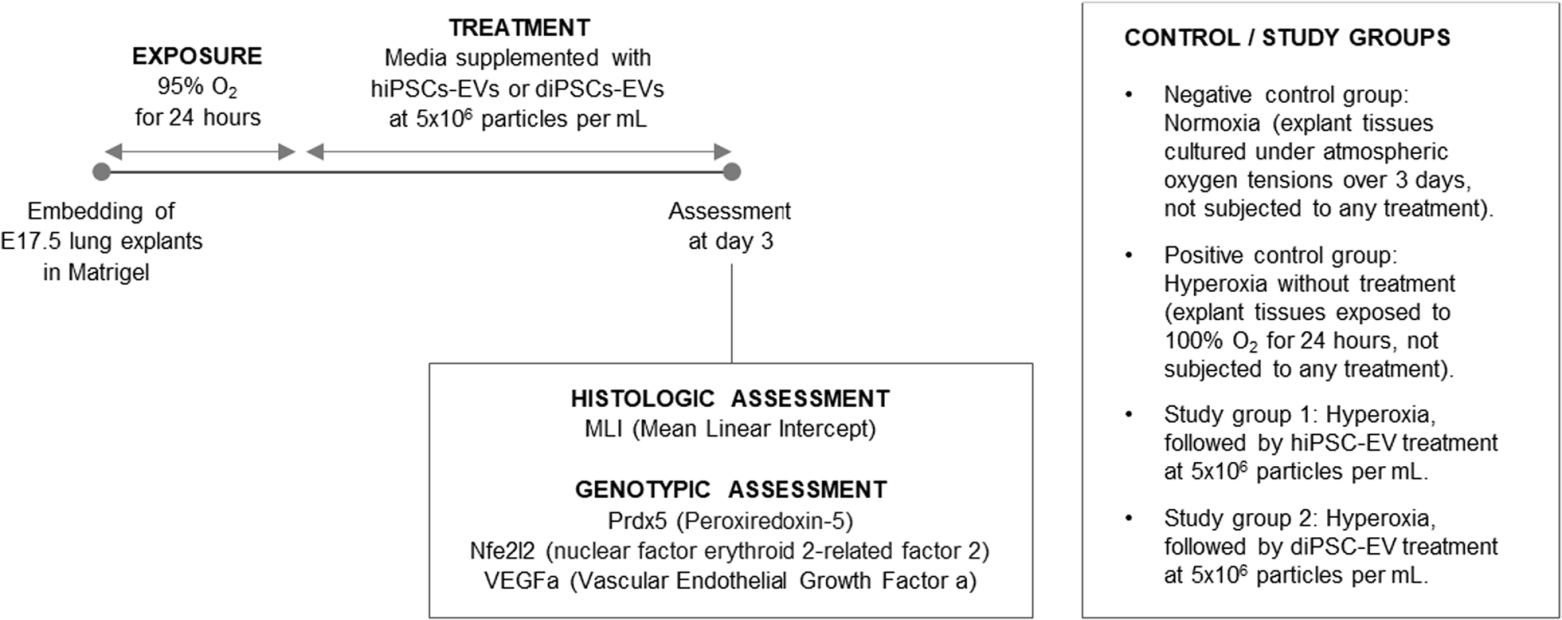
Experimental design. Fetal pups were randomly assigned to one of the 4 listed groups. The principal investigator was aware of the group allocation at different stages of the experiment (no blinding was performed)

Following 24 h of hyperoxia exposure, the plates were taken out of the modular incubator chamber, and the media below the membrane inserts was replaced and supplemented with either hiPSC-EVs or diPSC-EVs at 5 × 10^6^ particles per mL of media. Particle abundance in the EV preparations was determined by NTA. All samples were maintained during the treatment phase for 48 h at 37 °C, in 21% O_2_ and 5% CO_2_. The experimental design is outlined in Fig. 1. Controls included explants that remained in 21% O_2_ since their collection, and explants that were exposed to hyperoxia but left untreated. Histologic and genotypic assessment was performed on day 3 of in vitro culture.

#### Histologic assessment

Three samples from each group (normoxia, hyperoxia without treatment, hyperoxia followed by hiPSC-EV treatment, and hyperoxia followed by diPSC-EV treatment) were assessed histologically on day 3. Tissues were first vacuum-inflated to preserve their architecture during sectioning. Briefly, lung explants were transferred into labeled Eppendorf tubes filled with 10% formalin. The tubes were then placed in a vacuum desiccator, and negative pressure was applied for 20 min. This degassing technique has been reported in the literature [36, 37], and results in homogeneous lung inflation.

Following their inflation, tissues were embedded in OCT (Optimal Cutting Temperature compound, *Fisher Healthcare*) and stored at − 80 °C until sectioning. 10 µm slices were sectioned using a LEICA-CM3050S Cryostat, fixed in methanol/acetone mixture, stained with Hematoxylin and Eosin (H&E), and imaged on a Zeiss Observer inverted microscope at 10× magnification. Brightfield images were acquired, then processed stereologically using ImageJ software. Briefly, an 80-point grid system was applied to each image, and the following data were extracted: number of points hitting nonparenchymal (vascular or bronchial) structures, number of points hitting septal surfaces, and number of intercepts between septal structures and grid lines. The Mean Linear Intercept (MLI) was then derived from this data, as described in the literature [38].

Immunohistochemistry was performed on additional tissue sections to detect alterations in vasculature structure. The markers used were *DAPI* (4’,6-diamidino-2-phenylindole, for nucleic acids staining) and *PECAM* (platelet and endothelial cell adhesion molecule, for endothelial cell staining). Stained sections were also imaged on a Zeiss Observer inverted microscope at 10× magnification.

#### Genotypic assessment

Gene expression was assessed on day 3 using qRT-PCR. Tissues were lysed in buffer RLT (*Qiagen*), homogenized using a Bead Mill (*Fisher Scientific*), then centrifuged at 20,000× *g* for 3 min. The supernatant was then collected and stored at − 80 °C until RNA was extracted. An RNeasy Kit (*Qiagen*) was used for RNA isolation. RNA quality and concentration were checked with a Nanodrop One Spectrophotometer (*ThermoFisher*). Following DNAse treatment (*BioRad*) and reverse transcription (T100 Thermal Cycler, *BioRad*) PCR reactions were prepared, using the following primers: *Prdx5* (peroxiredoxin-5, *BioRad*), *Nfe2l2* (nuclear factor erythroid 2-related factor 2, also known as NRF2, *BioRad*), and *VEGFa* (vascular endothelial growth factor A, *BioRad*). The housekeeper *GAPDH* (*BioRad*) was used as the internal control gene. PCR reactions were run using a CFX96 Real-Time System (*BioRad*), and results processed with *BioRad* CFX Maestro software.

### Statistical analysis

Sample size was based on our initial observations that, following hyperoxia exposure, hiPSC-EVs, added to the medium at a concentration of 5 × 10^6^ particles/mL for 48 h, ameliorate airspace enlargement by around 30%. To achieve a study power of 80% with 5% significance for detecting a true improvement, a minimum of three samples per group were required. For statistical analysis, we used one-way Analysis of Variance (ANOVA) and Sidak Multiple Comparison Test (GraphPad). A *P*-value ≤ 0.05 was considered significant.

EV proteomic data was analyzed using Scaffold Q+ as described above. Differentially expressed proteins were determined by applying Mann–Whitney Test with unadjusted significance level *p* ≤ 0.05.

## Results

### EV characterization

**Fig. 2:**
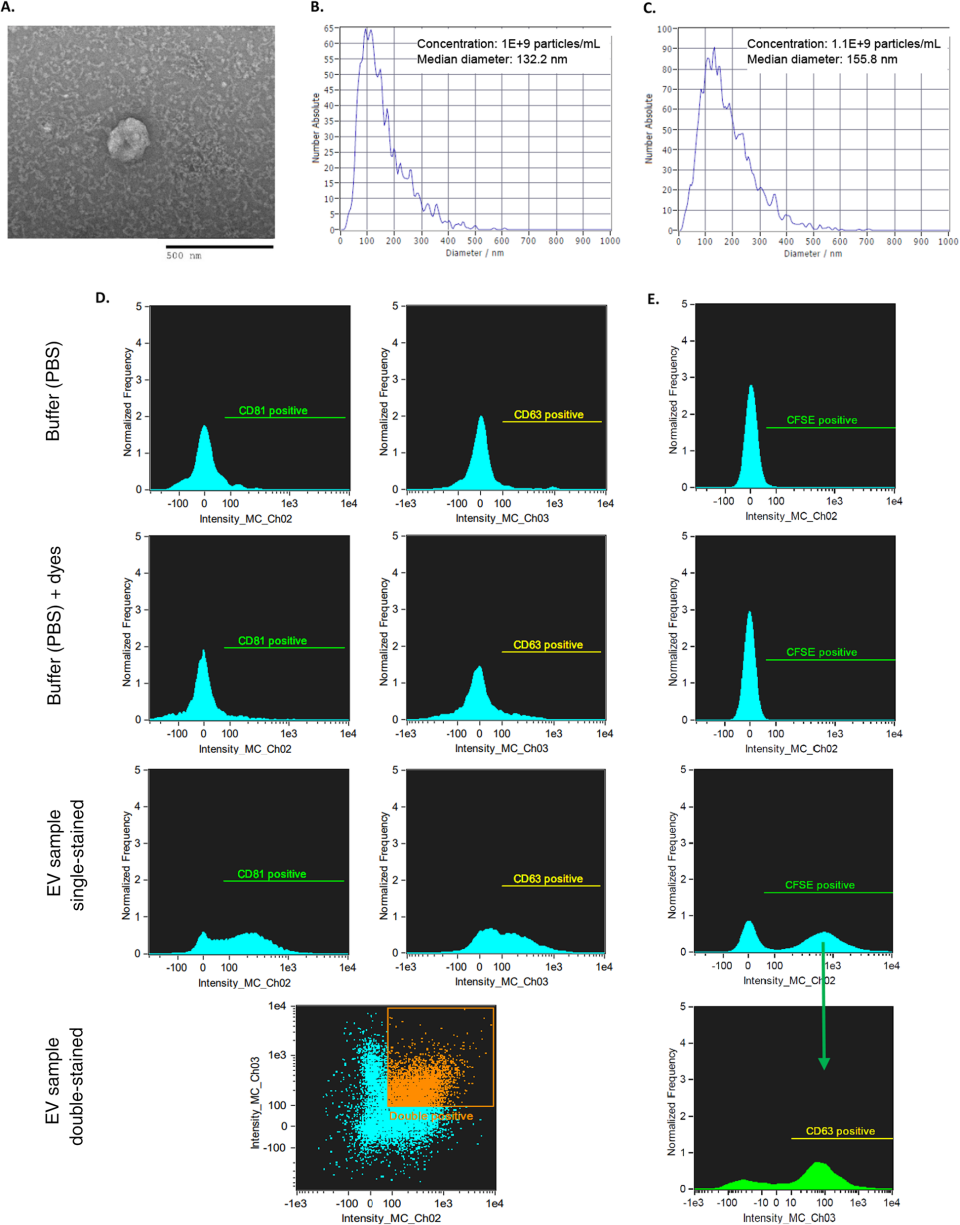
EV characterization. **A** Single EV visualized as a donut-shaped particle on TEM. **B/C.** Size distribution of hiPSC-EVs (**B**) and diPSC-EVs (**C**) as measured by Nanoparticle Tracking Analysis (NTA). **D/E.** Imaging flow cytometry (negative controls and hiPSC-EVs). Results of co-staining with antiCD81-FITC (Ch02) and antiCD63 (Ch03) are presented in **D**. EV gating with CFDA-SE (Ch02) followed by co-staining with antiCD63 (Ch03) is shown in **E**

EVs were visualized on TEM as single non-aggregating round, cup-, or donut-shaped particles (Fig. 2A). The cup and donut shapes result from air-drying the samples during their preparation for TEM, which causes the central soft part of the vesicle to collapse [39–41].

EV concentration and size distribution were determined by NTA. The isolated hiPSC-EVs had a median diameter of 132 nm with a standard deviation of 84 nm. diPSC-EVs isolated using the same technique, were slightly larger with a median diameter of 155 nm and a standard deviation of 95 nm. EV concentration in both samples was around 1 × 10^9^ particles per mL (Fig. 2B, C).

On IFC, EVs were detected as particles with low-scatter intensity given their low refractive index; the population acquired was 67%-positive for CD81, 49%-positive for CD63, and 34%-double-positive (Fig. 2D). Additional gating was performed using CFSE dye to focus on intact EVs. Intact vesicles were 70% positive for CD63 (Fig. 2E). Acquired small particles with low-scatter intensity but negatively staining for CFSE may represent contaminants or protein aggregates. Proteomic profiling by Mass Spectrometry showed indeed the presence of some non-vesicular contaminants in the sample, such as ribosomal proteins.

### Histologic and genotypic changes following in vitro exposure of fetal lung explants to hyperoxia

**Fig. 3:**
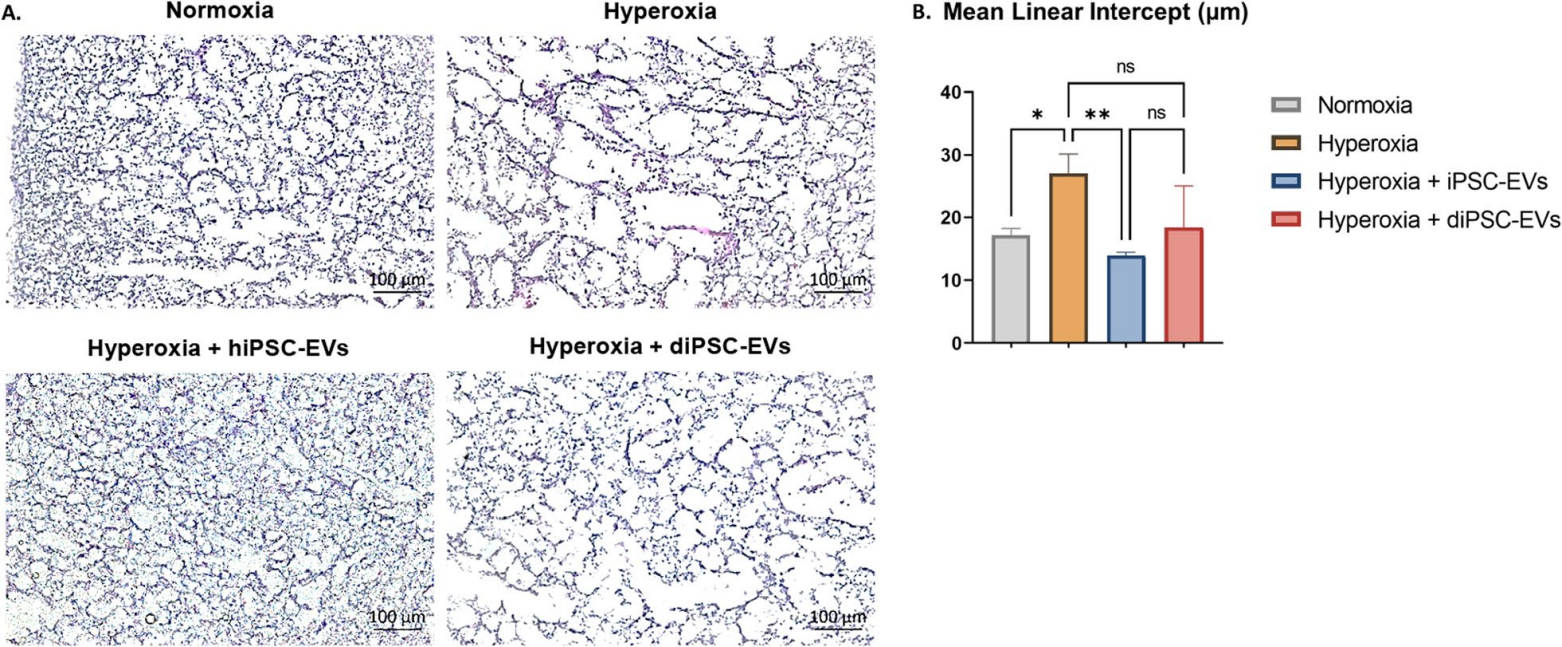
Effect of hiPSC-EV and diPSC-EV administration on lung architecture. **A** Imaging of sections stained with H&E: hyperoxia-induced airspace enlargement is much improved following treatment with hiPSC-EVs; less significant improvement is noted with diPSC-EVs. **B** Comparative analysis of mean linear intercepts in the different groups (n = 3 in each group; and, for each tissue, 4 images were analyzed). One-way ANOVA was used for statistical analysis. *p ≤ 0.05. **p ≤ 0.01

**Fig. 4:**
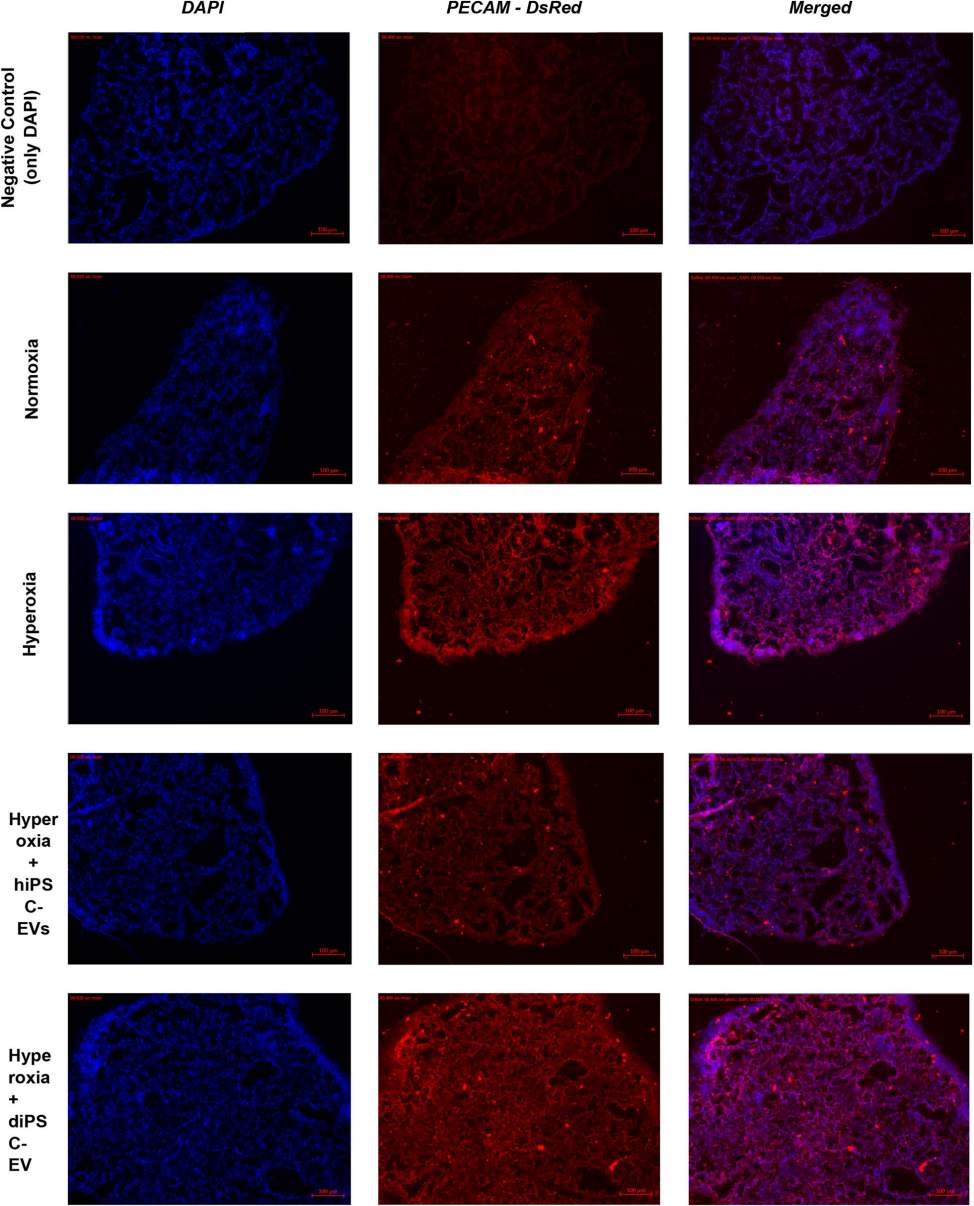
Effect of hyperoxia exposure and EV treatment on tissue vasculature. No differences were detected between the four groups (normoxia, hyperoxia, hyperoxia + hiPSC-EVs, hyperoxia + diPSC-EVs). A negative control tissue stained only with DAPI is shown

**Fig. 5:**
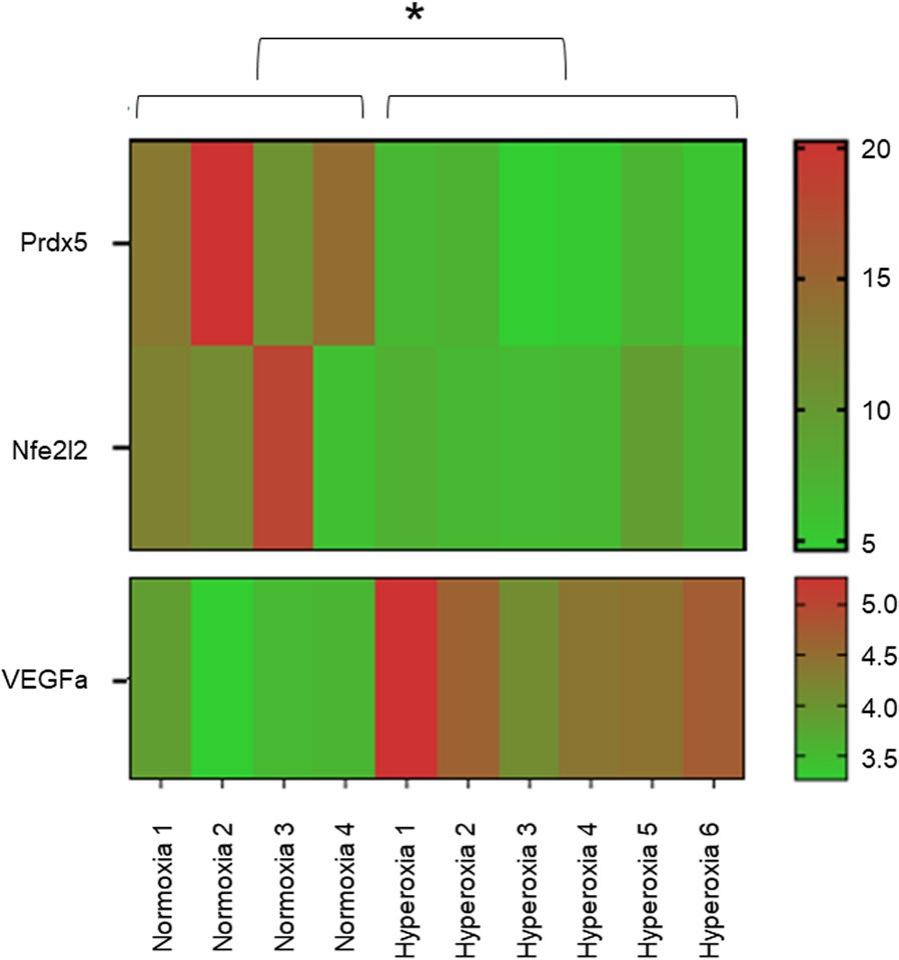
Heatmap representing *Prdx5*, *Nfe2l2*, and *VEGFa* gene expression in normoxic and hyperoxic samples, based on qRT-PCR results. ΔCq values are presented. A higher Cq value implies a lower gene expression. The anti-oxidant genes *Prdx5* and *Nfe2l2* were differentially upregulated in hyperoxic samples (*p* = 0.0175 for *Prdx5* expression, *p* = 0.0298 for *Nfe2l2* expression). *VEGFa* was downregulated following hyperoxia exposure (*p* < 0.0001)

Exposure of E17.5 lung explants to 95% O_2_ for 24 h resulted in a significant increase in the MLI from 17.18 ± 0.87 µm to 27.01 ± 2.56 µm, indicating alveolar damage. Airspaces appeared enlarged and disrupted on stained sections (Fig. 3A). PCR analysis showed a statistically significant upregulation of the antioxidant genes *Prdx5* (490-fold change, *p* = 0.0175) and *Nfe2l2* (27-fold change, *p* = 0.0298). Although no overt changes in tissue vasculature were noted on immunohistochemistry (Fig. 4), *VEGFa* expression was significantly reduced (0.5-fold change, *p* < 0.0001) in hyperoxia-exposed samples, relative to tissues maintained in normoxia (Fig. 5).

### hiPSC-EV and diPSC-EV administration improves histologic outcomes in fetal explants exposed to hyperoxia

Tissues treated with 5 × 10^6^ particles per mL of hiPSC- or diPSC-EVs over 48 h following injury were less impacted by hyperoxia (Fig. 3A, B). Average MLI was reduced by 48% with hiPSC-EVs (from 27.01 ± 2.56 µm to 13.95 ± 0.4 µm), and by 32% with diPSC-EVs (from 27.01 ± 2.56 µm to 18.40 ± 5.38 µm). Statistical significance was only reached with hiPSC-EVs (*p* ≤ 0.01).

### diPSC-EV administration upregulates anti-oxidant gene expression and VEGFa expression in fetal explants exposed to hyperoxia

**Fig. 6:**
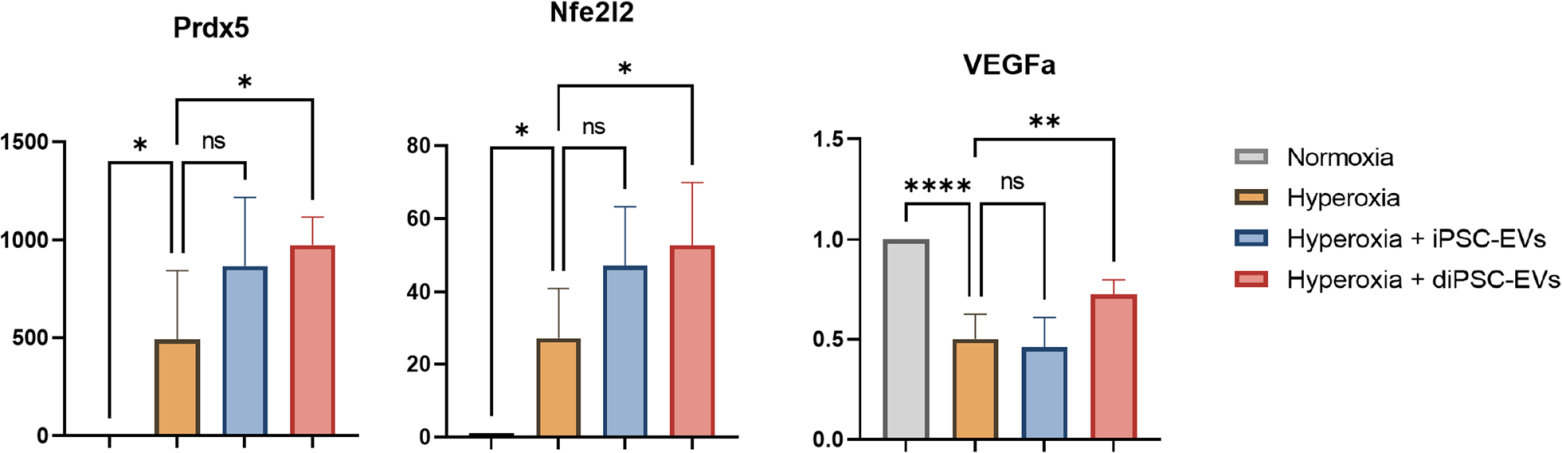
Effect of hiPSC-EV and diPSC-EV administration on *Prdx5*, *Nfe2l2* and *VEGFa* gene expression, based on qRT-PCR results. Treatment with diPSC-EVs following hyperoxia exposure induced a significant upregulation of the antioxidant genes *Prdx5* and *Nfe2l2* expression, and also an increase in *VEGFa* expression. The effects of hiPSC-EVs on the expression of these genes were less pronounced. *p ≤ 0.05. **p ≤ 0.01. **p ≤ 0.0001

Treatment with hiPSC-EVs and diPSC-EVs induced a more pronounced upregulation of the antioxidant genes *Prdx5* and *Nfe2l2* (Fig. 6 and Additional file 1: Fig. 3). *Prdx5* expression increased from 490-fold in hyperoxic samples (relative to normoxic samples) up to 868-fold with hiPSC-EV treatment (*p* = 0.0587), and up to 975-fold with diPSC-EV treatment (*p* = 0.0135). Similarly, *Nfe2l2* expression increased from 27-fold in hyperoxic samples (relative to normoxic samples) up to 47-fold with hiPSC-EV treatment (*p* = 0.0663), and up to 53-fold with diPSC-EV treatment (*p* = 0.0164). Statistical significance was reached only with diPSC-EV treatment for both genes.

*VEGFa* expression, which was found to be downregulated in hyperoxic samples in comparison to normoxic samples, significantly increased with diPSC-EV treatment (from a 0.5-fold to a 0.72-fold change, *p* = 0.0047). *VEGFa* expression remained unchanged with hiPSC-EV treatment (Fig. 6 and Additional file 1: Fig. 3).

### hiPSC-EVs and diPSC-EVs exhibit differing proteomic profiles in terms of their anti-inflammatory, anti-oxidant, and regenerative protein content

To better understand the therapeutic effects of hiPSC- and diPSC-EVs, their proteomic profiles were analyzed using mass spectrometry. In total, 659 proteins were identified. While the two EV groups shared a significant number of membrane and luminal proteins, some proteins were unique to each population or were more represented in one than the other. We were particularly interested in proteins involved in anti-inflammatory, anti-oxidant, and regenerative pathways, and in the processes of alveolarization and lung angiogenesis. The number of replicates used for analysis were 3 replicates for hiPSC-EVs and 2 replicates for diPSC-EVs.

**Fig. 7:**
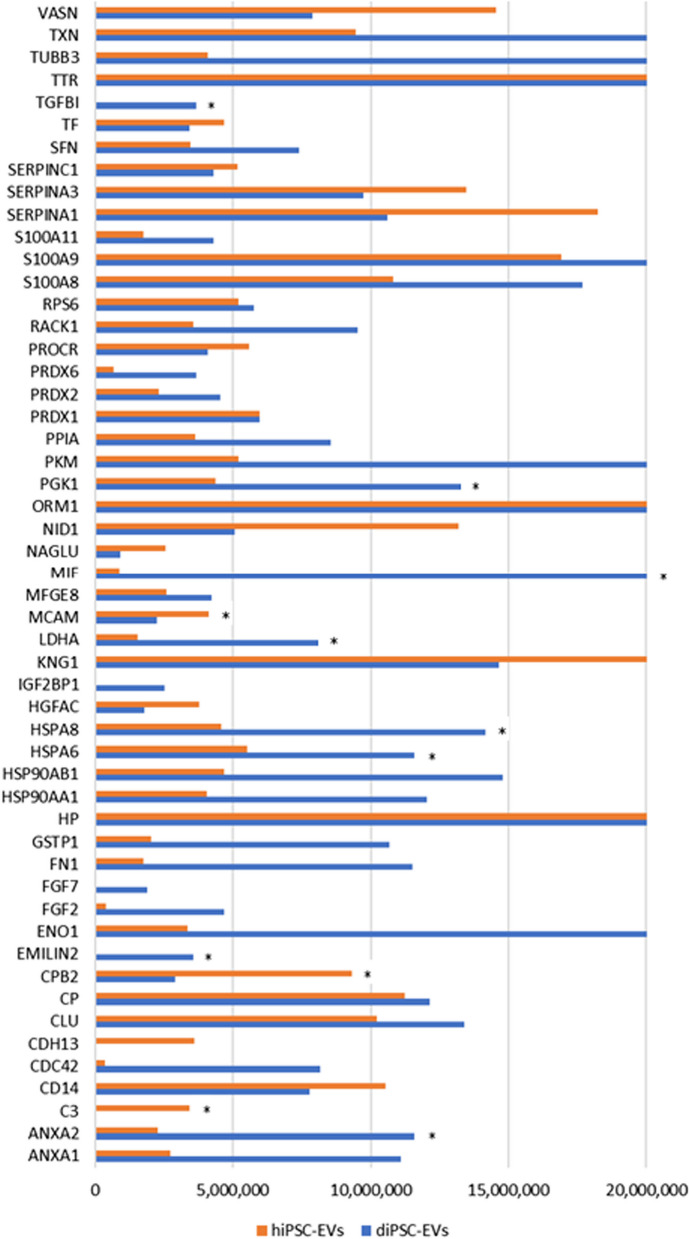
Comparative analysis of the proteomic profiles of hiPSC-EVs and diPSC-EVs. The graph is a bar chart representing the Normalized Average Precursor Intensity of proteins implicated in anti-oxidant, anti-inflammatory and regenerative pathways. Statistically significant differences between hiPSC-EV and diPSC-EV proteomic content are labeled with * (Mann–Whitney test, *p* < 0.05)

**Fig. 8:**
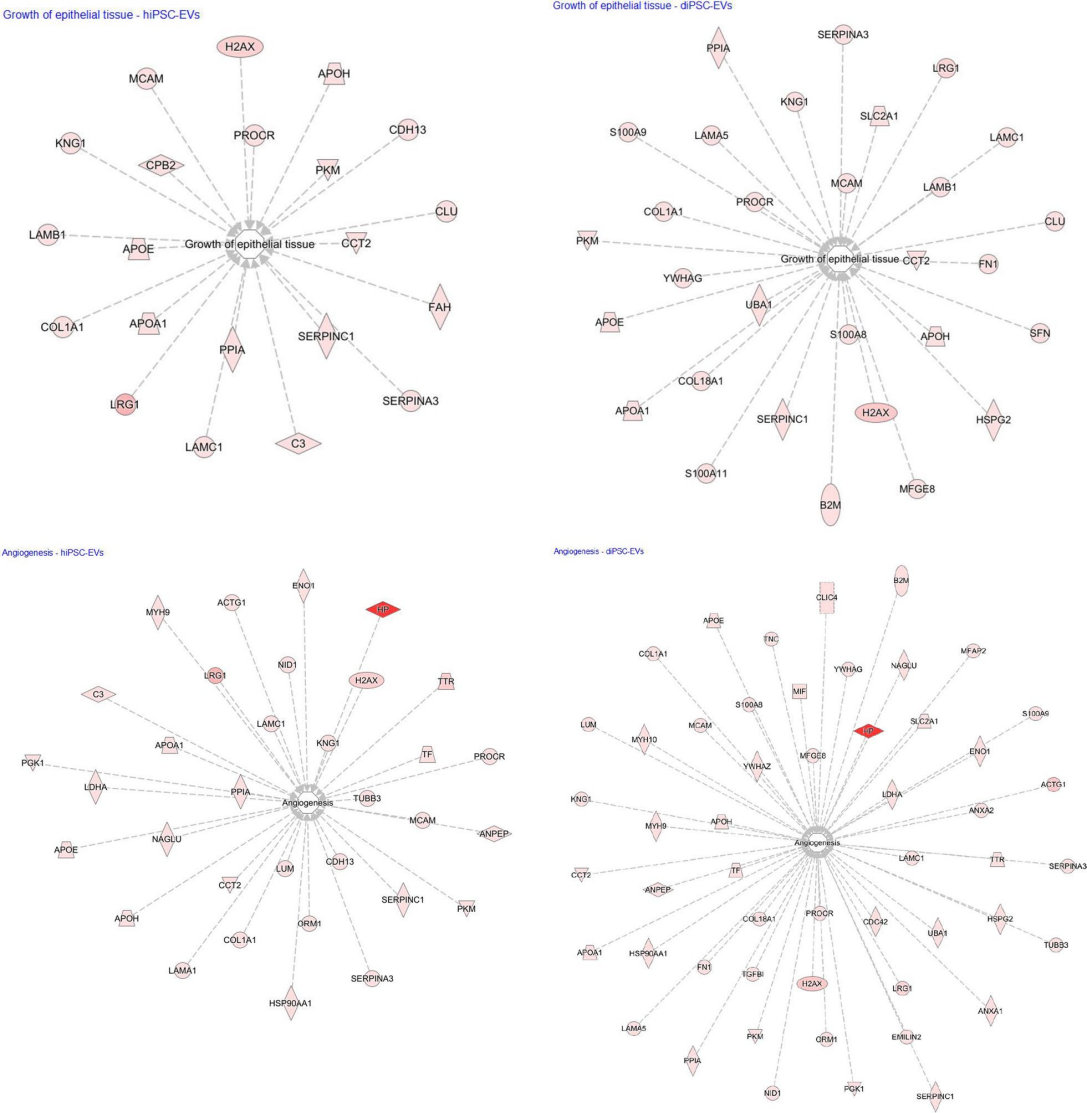
EV molecules contribute to the growth and regeneration of epithelial and endothelial tissue. Protein connections were derived from data analysis in IPA (*Qiagen*). Both hiPSC-EVs and diPSC-EVs are enriched in factors that drive the processes of alveolarization and angiogenesis. The intensity of the red color inside each protein box is directly proportional to the abundance of this protein in EVs. For example, haptoglobin (HP) is highly expressed in both hiPSC-EVs and diPSC-EVs, more so than other molecules

**Fig. 9:**
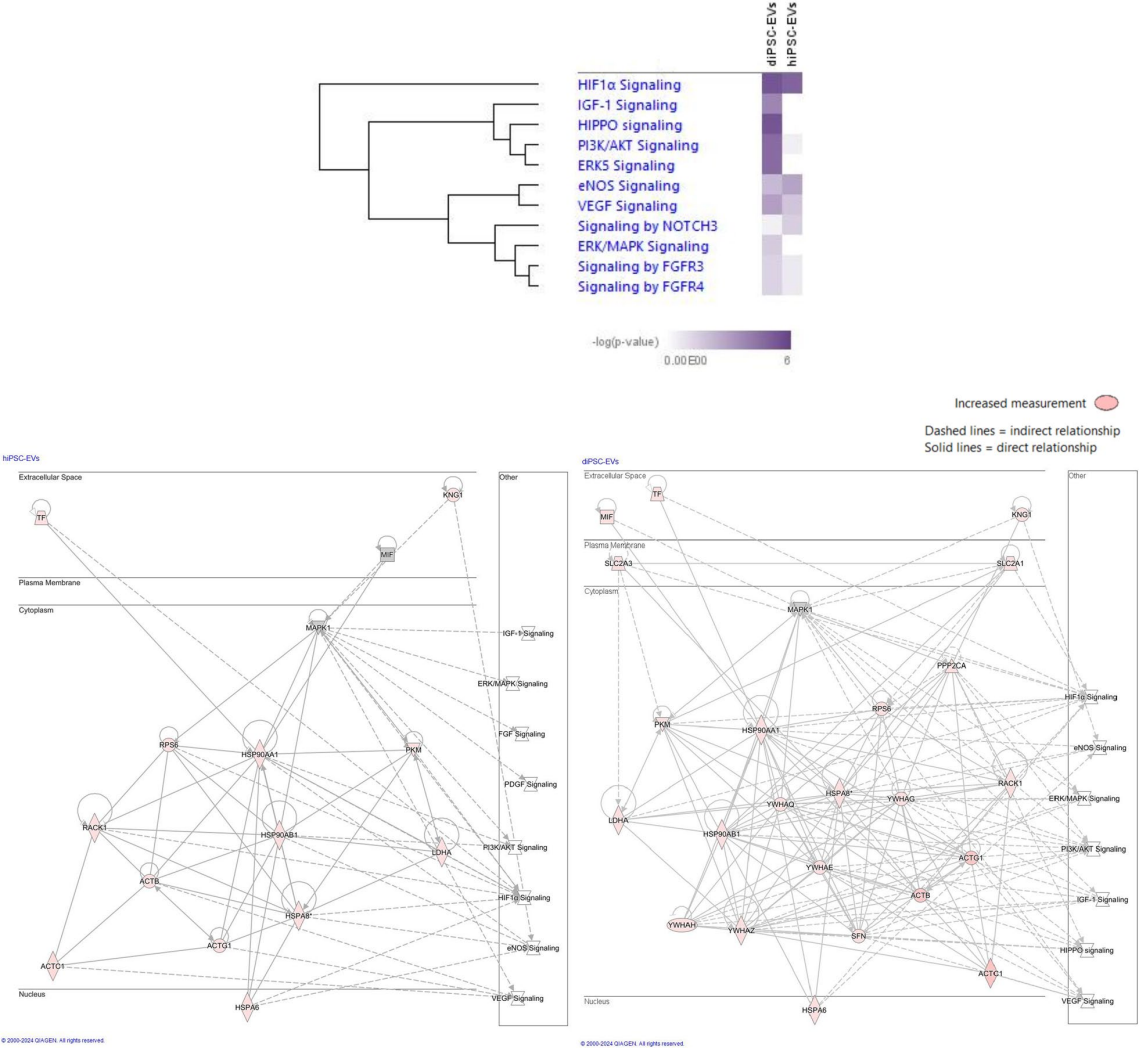
Matching EV proteomics to pathways implicated in alveolarization and angiogenesis. Pathways and connections were derived from data analysis in IPA (*Qiagen*). Several signaling pathways implicated in the processes of alveolarization and angiogenesis were identified. The heatmap represents the abundance, within hiPSC-EVs and diPSC-EVs, of proteins connected to each of these pathways. The darker the color, the higher is the protein abundance

Some anti-inflammatory, anti-protease and regenerative proteins were found to be more expressed in hiPSC-EV samples (Fig. 7). For instance, hepatocyte growth factor activator (*HGFAC*) was 2.1-times more abundant in hiPSC-EVs than diPSC-EVs. Other examples include alpha-1-antitrypsin (*SERPINA1*, 1.6-fold more abundant in hiPSC-EVs, *p*-value 0.012) and alpha-1-antichymotrypsin (*SERPINA3*, 1.4-fold more abundant in hiPSC-EVs, *p*-value 0.28). On the other hand, diPSC-EV samples were more enriched than hiPSC-EVs in anti-oxidant factors such as glutathione transferase (*GSTP1*, 5.2-fold change, p-value 0.063) and macrophage migration inhibitory factor (*MIF*, 24-fold change, p-value 0.027). *MIF* is also known to up-regulate *VEGFa* expression and activate multiple angiogenic pathways (Figs. 8 and 9).

Upon further analysis using *Reactome* Tool and *IPA* Software, proteins identified in both hiPSC-EVs and diPSC-EVs were found to be implicated in multiple signaling pathways known to drive the processes of alveolarization and angiogenesis during normal development and in response to injury. Examples include *HIF1α*, *IGF1*, *NOTCH*, *Hippo*, *FGF*, *PDGF*, *VEGF* and its downstream signaling pathways *Pl3*/*Akt, ERK/MAPK* and *eNOS* activation (Figs. 8 and 9). Comparative analysis showed that diPSC-EVs are more enriched in proteins implicated in *VEGF* signaling (1.7-fold more than hiPSC-EVs) and *Pl3/Akt* signaling (7.8-fold more than hiPSC-EVs), while hiPSC-EVs carry more factors involved in *NOTCH* signaling (3.1-fold more than diPSC-EVs) and *eNOS* signaling (1.3-fold more than hiPSC-EVs) (Fig. 9).

## Discussion

### hiPSCs as stem cell source of EVs: benefits and drawbacks

In the past few years (2018–2023), EVs derived from mesenchymal stem cells (MSCs) have gained popularity in animal BPD studies and were shown to be effective in improving structural and functional lung outcomes following hyperoxic exposure [42, 43]. Heterogeneity exists however among these studies in terms of cell source, EV isolation and characterization techniques, model used, route of EV administration, timing and dosage. To the best of our knowledge, only one previous study evaluated the effectiveness of hiPSC-derived EVs in attenuating hyperoxia-induced lung changes, but was limited by the technique used to isolate EVs [5]. Our study improves upon that approach, by using methods that allow for the recovery of a highly specific EV population (ultrafiltration combined with size-exclusion chromatography). We also used a robust approach for EV characterization in accordance with the recommendations of MISEV2018 (Minimal Information for Studies of Extracellular Vesicles) [23]. Our samples derived from a single hiPSC cell line and a single diPSC cell line, which reduced the risk of batch-to-batch variability.

hiPSC-EVs are interesting to evaluate as a potential intervention for premature lung disease for multiple reasons. First, they are easy to generate from adult somatic cells. Reprogramming kits have been well optimized, and cell sources are easy to access via skin, blood, or urine. Autologous delivery is also possible. Second, hiPSCs exhibit the same level of pluripotency and potential for self-renewal as embryonic stem cells without carrying their ethical concerns. hiPSCs are thus theoretically more potent than MSCs, and studies have shown they have more immunomodulatory benefits than MSCs [44]. Third, previous proteomic studies have shown that hiPSC-EVs carry proteins implicated in *FGF2*, *VEGF*, *IL-4* and *EGFR* pathways more abundantly than MSC-EVs [45]. These pathways are all known to play a significant role in lung tissue regeneration and repair following injury. Finally, clinical trials using hiPSCs are more cost-effective than those involving MSCs [44].

Nonetheless, hiPSC use is not without limitations. Their major drawback is their potential immunogenicity and tumorigenicity. The differentiation of hiPSCs into distal lung phenotypes (diPSCs) before their administration to disease models was shown to mitigate the risk of teratogenicity [15]. diPSCs are intended to resemble alveolar epithelial cells type II. These cells are also known to have immunomodulatory effects and are capable of proliferating and differentiating into alveolar epithelial cells type I following injury.

Another strategy that might help reduce both risks of immunogenicity and teratogenicity is the use of EVs instead of whole cells. Major histocompatibility complex (MHC) genes, known to play a pivotal role in the adaptive immune system, are less abundant in a cell secretome than on its surface [46–48]. Teratogenicity is also less of a concern since, unlike whole cells, secreted particles cannot self-replicate. The effectiveness of EVs in repairing premature lung injury, whether derived from hiPSCs or diPSCs, is the object of this study.

### The fetal lung explant model: pros and cons

Our lung explant model recapitulates the clinical setting of extreme prematurity and high oxygen exposure leading to the disruption of normal lung development. The structural and functional units of the lung are kept intact as opposed to other in vitro models utilizing cells alone [31, 49–51]. To mimic the arrest of lung morphogenesis at the late canalicular or early saccular stage, we developed an E17.5 murine lung explant model which we exposed to hyperoxia in vitro. Embedding the tissue in Matrigel at an air–liquid interface and in the presence of FGF-2, FGF-7 and FGF-10 preserved tissue viability throughout the experiment. Twenty-four hours of 95% O_2_ exposure in vitro were sufficient to recapitulate some of the histologic and genotypic hallmarks of the disease, including airspace disruption, decreased *VEGFa* expression [52], and upregulation of cytoprotective anti-oxidant genes *Prdx5* and *Nfe2l2* to counteract the oxidative stress [53].

A major drawback of our model is its inability to mimic the in vivo upregulation of some inflammatory markers such as *TGF-b1*, *NF-κB1* and *IL6* by hyperoxia exposure [10]. The expression of these genes remained unchanged in our explants, as shown in Additional file 1: Fig. 4; thus, they were not selected for this EV study. We postulate that, for these genes to be upregulated, inflammatory cells need to be recruited and activated (macrophages, activated B cells). A potential adjustment for future studies would be co-culturing the in vitro model with inflammatory cells; hyperoxia exposure might then result in the upregulation of these genes.

Our model also failed to demonstrate alterations in tissue vasculature structure following hyperoxia exposure. Nonetheless, we detected a downregulation of *VEGFa* expression with qRT-PCR, which indicates that changes have already occurred at a genotypic level. *VEGFa* is the primary driver of angiogenesis. We postulate that the duration of exposure to injury (24 h in our study) and the total duration of tissue culture (3 days in our study) might not have been sufficient to induce structural alterations in vasculature. Lengthening exposure time in future studies and maintaining tissues in culture for a longer period of time before their assessment (provided they remain viable) will allow to test this hypothesis.

### hiPSC- and diPSC-EV based treatment: promises

Following hyperoxia exposure, lung architecture was improved with EV treatment, most significantly with hiPSC-EVs. Of note, we tested two hiPSC-EV concentrations: 2.5 × 10^6^ and 5 × 10^6^ particles/mL. The improvement in lung architecture was more robust with the latter dose, which was then extrapolated for use in diPSC-EV treated samples. A larger sample size or a higher EV dose might be needed to achieve statistical significance with diPSC-EVs. Although functional outcomes are beyond the scope of this in vitro study, the improvement in lung architecture and alveolar septation would imply a greater area for gas exchange and would predict an improvement in lung function in vivo.

Interestingly, diPSC-EVs seem to have a great pro-angiogenic potential. They are enriched in proteins involved in angiogenic pathways and their administration resulted in the up-regulation of *VEGFa* expression following hyperoxia exposure. This effect could be in part explained by the large abundance of macrophage migration inhibitory factor (*MIF*) in diPSC-EVs, more so than in hiPSC-EVs. Several studies have reported a strong correlation between *MIF* and *VEGF* expression. Exogenous *MIF* has been shown to promote angiogenesis by inducing a dose-dependent upregulation of *VEGF* [54, 55].

diPSC-EVs are also effective in up-regulating the expression of anti-oxidant genes *Prdx5* and *Nfe2l2*. Therefore, they may play a better cytoprotective role if administered during the period of hyperoxia exposure rather than at a later time following the injury. *Prdx5* encodes for a mitochondrial protein capable of reducing reactive oxygen species. *Nfe2l2* encodes for a transcription factor that promotes the expression of antioxidant enzymes which help attenuate the oxidative stress. The upregulation of these genes could also be attributed to the large abundance of *MIF* in diPSC-EVs. *MIF* is known to be an anti-oxidant cytokine. When human lung endothelial cells were *MIF*-silenced, they failed to upregulate *Nfe2l2* expression following oxidative stress injury [56, 57]. Knock-out studies are still needed to correlate any identified EV protein to the suggested function. Once an EV-associated component is linked to a specific function, further characterization of the topology of this component (luminal or surface) is recommended as per MISEV2018 guidelines [23].

Proteins identified in hiPSC-EVs and diPSC-EVs are implicated in key pathways known to drive the processes of alveolarization and angiogenesis during normal development and in response to injury: *HIF1α*, *IGF1*, *NOTCH*, *Hippo*, *FGF*, *PDGF*, *VEGF*, *Pl3*/*Akt, ERK/MAPK* and *eNOS* activation (Fig. 9).

hiPSC-EVs are particularly enriched in factors involved in *NOTCH* signaling, 3.1-fold more abundantly than diPSC-EVs. *NOTCH* signaling is extremely important in the canalicular and saccular stages of lung development as a primary determinant of cellular fate. The differentiation of distal epithelial cells into pneumocytes I and II, and proximal epithelial cells into multi-ciliated or secretory cells, is largely mediated by *NOTCH* receptors [58–60]. When cellular balance is lost during injury, *NOTCH* activation plays an important role in regeneration and repair by regulating the commitment of cells towards one type or another [61, 62]. The enrichment of hiPSC-EVs in molecules capable of regulating the *NOTCH* signaling pathway holds promise for the future use of EV-based therapies in the treatment of lung diseases of prematurity. Other identified pathways, such as *Hippo* signaling, *FGF* activation and *PDGF* signaling, are also known to regulate the alveolarization process during normal development and following injury [63–65].

Multiple key angiogenic pathways were identified, most importantly *VEGF signaling*, and its downstream pathways, *Pl3*/*Akt, ERK/MAPK* and *eNOS* activation. diPSC-EVs are particularly enriched in proteins implicated in *VEGF* signaling (1.7-fold more than hiPSC-EVs) and *Pl3/Akt* signaling (7.8-fold more than hiPSC-EVs), which explains their ability to upregulate *VEGF* expression in our explant model. Similarly, a recent study showed that EVs isolated from an immortalized airway cell line were *effective* in promoting the survival of endothelial cells, specifically via the activation of *VEGFa* binding receptor, *VEGFR2* [66]. This pathway is known to cross-talk with other angiogenic pathways, such as *ERK1*/*ERK2* activation, *Pl3*/*Akt*, *eNOS,* and *HIF-α* hydroxylation, all identified in our EV proteomic analysis. It is also important to note that, aside from its angiogenic potential, *VEGF* is a key driver of alveolarization during lung development and following injury [52].

Diving deeper into the role of stem cell-derived EVs in the regulation of these pathways will help engineer EV populations targeted towards specific therapeutic applications where a specific pathway needs to be activated. This will pave the way for an individualized approach to prevent or treat diseases that can have multiple phenotypes, such as lung disease of prematurity.

### Limitations and challenges

**Fig. 10:**
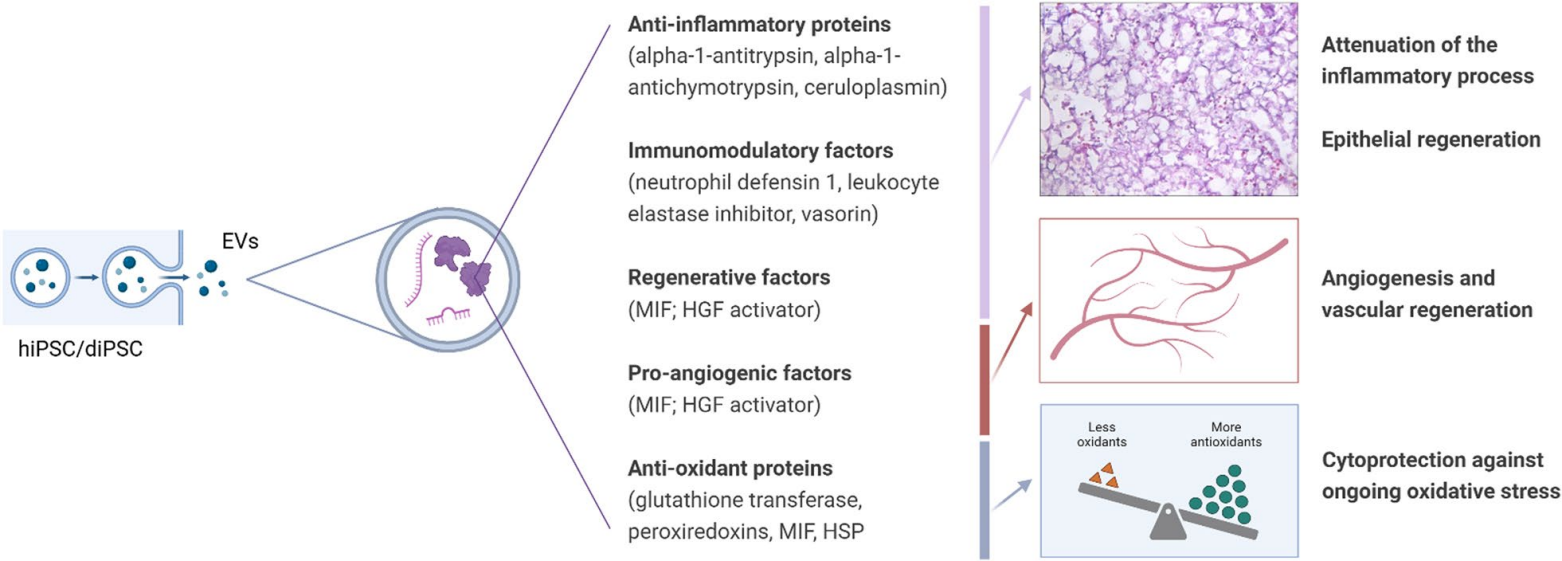
Potential therapeutic benefits of hiPSC-EVs and diPSC-EVs in hyperoxia-induced lung injury. While the exact mechanism of action of these EVs remains unclear, this diagram summarizes some of the hypotheses generated from EV proteomic data. Abbreviations: HGF: hepatocyte growth factor—HSP: heat shock protein—MIF: macrophage migration inhibitory factor

While the exact mechanism of action of hiPSC- and diPSC-EVs remains unclear, we were able to generate multiple hypotheses using EV proteomic findings (Fig. 10). Nonetheless, several challenges were encountered during this study, and we acknowledge the presence of multiple limitations.

First, the small size of the fetal murine lung explant makes it easily susceptible to mechanical injury during collection and sectioning. Unwanted micro-tears in the tissue may confound the histologic results. Although precautions were taken to exclude tissues thought to be mechanically injured during the experimental process, the distinction was not always clear. Furthermore, in vitro studies, like ours, are unable to recapitulate the complexity of the pathophysiologic response to injury that occurs in extreme preterm infants. Better models are needed to resolve these issues, including standardized 3D-printed distal preterm lung models and larger in vivo animal models that can survive premature birth. Nonetheless, our explant model provides proof of concept for the use of EV-based therapies in the treatment of hyperoxia-induced lung injury. Now that primary methods have been established, a larger in vivo model will be used to further confirm our findings. Techniques such as microcomputed imaging to assess lung tissues would preserve their architecture much better than current sectioning techniques and will be used in future studies.

Second, the differentiation process of hiPSCs into alveolar-like cells was carried out in a two-dimensional (2D) culture environment. The presence of some pro-inflammatory proteins in our diPSC-EV samples (such as transforming growth factor beta induced protein *TGFBI* (Fig. 7) was noted and might have resulted from intrinsic alterations during the 2D-differentiation process. A three-dimensional (3D) culture system would better recapitulate the physiologic differentiation of progenitor cells into Type II pneumocytes [67, 68]. A much larger EV yield would also be obtained with 3D cell cultures [69, 70].

Third, although our EV isolation technique resulted in a relatively highly specific population, contaminants were still present in the sample. When NTA was performed on PBS samples running through the chromatography column, nanoparticles were detected, as shown in Additional file 1: Fig. 5. These contaminants are likely inherent to the column itself and represent only 2.5% of the total particles detected by NTA in EV samples. They did not seem to have a harmful effect on our lung explant model. Nonetheless, their safety remains a concern for clinical translatability and will need to be investigated further in future experiments.

Additionally, it is important to note that EVs carry, not only proteins, but also nucleic acids (mRNAs and miRNAs). There is evidence in the literature that EVs’ therapeutic effects are largely mediated by the genetic exchange that occurs with recipient cells [71–73]. EV genomic content was not studied in this project but might as well explain our promising findings. Thus, therapeutic effects cannot be solely attributed to proteomic data.

Lastly, EVs were stored at − 20 °C in PBS for 2–3 months before their usage. Characterization was performed prior to storage. Recent studies showed that these storage conditions may impair EV stability and reduce the concentration of intact vesicles in the sample [74, 75]. Storing EVs at − 80 °C in PBS supplemented with human albumin and trehalose seems to better preserve EV stability over time [75]. Regardless of the storage conditions, it would be advantageous to characterize the samples again immediately prior to their experimental use. Furthermore, transitioning towards future clinical applications requires a large-scale production of functional, homogeneous, and intact EVs. To reach this goal, several challenges still need to be addressed, including optimizing cell culture conditions to obtain a high EV yield, standardizing EV isolation and characterization techniques to ensure reproducibility, optimizing storage conditions to preserve bioactivity, and developing stringent regulations with measurable quality standards. EVs’ safety is also yet to be determined.

## Conclusion

In conclusion, this proof-of-concept study shows a potential role for hiPSC- and diPSC-EVs in attenuating lung changes associated with prematurity and oxygen exposure in a murine lung explant model. Future research using validated in vivo BPD models is needed to confirm these findings and to further understand the mechanisms behind the regenerative action of EVs. Functional outcomes and safety profiles are also yet to be determined. This study, despite its limitations, paves the way for a novel stem-cell free approach to prevent and/or treat BPD, and ultimately reduce the global burden of the disease.

### Supplementary Information

**Additional file 1: Figure 1** hiPSC characterization during the differentiation process, using flow cytometry. Prior to their differentiation, hiPSCs had over 95% positive expression for the pluripotency markers OCT4, SOX2, NANOG and TRA-1-60. At the Definitive Endoderm DE stage (day 3 of differentiation), cells expressed more than 80% positivity for CXCR4 and EPCAM. At the Anterior Foregut Endoderm AFE stage (day 5 of differentiation), 89% positivity for SOX17 and 73% positivity for FOXA2 were obtained. FOXA2 is a key marker of AFE while SOX17 marks the transition from DE to AFE. On day 25, cells had reached the distal lung phenotype stage (diPSCs). Their positivity for pluripotent markers has much decreased. Over 90% positivity for TTF1 and T1alpha, 33% positivity for AQP5 and 4% positivity for SP-C were obtained at this stage. **Figure 2** A. Schematic representation of the fetal lung explant model (Abbreviations: BM: basement membrane; FGF: fibroblast growth factor). B. Preserved viability of the explant model for up to 7 days of in vitro culture. Calcein and Ethidium Bromide were used for Live/Dead staining. Imaging was carried out using a Zeiss Observer inverted microscope. Over 85% of viability was maintained throughout the in vitro explant culture, validating its suitability for experimental use during this time period. To note that the study experiment was carried out over the first 3 days of culture. **Figure 3** *Prdx5*, *Nfe2l2* and *VEGFa* gene expression level relative to the internal control GAPDH (delta Cq), based on qRT-PCR. Refer to fold-change relative to the internal control and to the normoxia tissue in Fig. 6. The lower delta Cq is, the higher is the fold-gene expression. **Figure 4** Relative normalized expression of *IL6*, *NF-κB1* and *TGF-b1*, based on qRT-PCR results. These genes are known to be upregulated following hyperoxia injury in vivo, but no changes were observed in our in vitro model. **Figure 5** NTA of a PBS sample run through the chromatography column. This is a negative control sample. Nanoparticles were nonetheless detected and are likely to represent contaminants inherent to the column itself. These particles were much less abundant than those detected by NTA in EV samples (they represent only 2.5% of the total particles detected in EVs) and had more heterogeneous diameters (widespread distribution with large standard deviation). While these contaminants did not seem to have a harmful effect on our lung explant model, their safety remains a concern for clinical translatability and will need to be further investigated.

## Data Availability

The data supporting this article’s findings are available from the corresponding author upon reasonable request.

## Abbreviations

2D: Two-dimensional
3D: Three-dimensional
AFE: Anterior foregut endoderm
Akt: Protein kinase B
ANOVA: One-way analysis of variance
ATCC: American Type Culture Collection
BM: Basement membrane
BMP4: Bone morphogenetic protein
BPD: Bronchopulmonary dysplasia
CFDA-SE: Carboxyfluorescein diacetate succinimidyl ester
CXCR4: Chemokine receptor type 4, also known as fusin or CD184
DE: Definitive endoderm
diPSCs: HiPSCs differentiated into distal lung phenotype
DMEM: Dulbecco’s Modified Eagle Medium
E-: Embryonic day
EGFR: Epidermal growth factor receptor
eNOS: Endothelial nitric oxide synthase
EPCAM: Epithelial cell adhesion molecule, also known as CD326
ERK: Extracellular signal-regulated kinase
EVs: Extracellular vesicles
F12: Ham’s F12 medium
FGF: Fibroblast growth factor
FITC: Fluorescein isothiocyanate
FOXA2: Forkhead box A2
GSTP1: Glutathione transferase
H&E: Hematoxylin and eosin
HBSS: Hanks Balanced Salt Solution
HGFAC: Hepatocyte growth factor activator
HIF1α: Hypoxia inducible factor-1 alpha
hiPSCs: Human induced pluripotent stem cells
HSP: Heat shock protein
IACUC: Institutional animal care and use committee
IDEAS: Image data exploration and analysis software
IFC: Imaging flow cytometry
IGF1: Insulin growth factor-1
IL-4: Interleukin-4
IL-6: Interleukin-6
IPA: Ingenuity pathway analysis
LCMS: Liquid chromatography–mass spectrometry
LP: Lung progenitor
MAPK: Mitogen activated protein kinase
MHC: Major histocompatibility complex
MIC: Modular incubator chamber
MIF: Macrophage migration inhibitory factor
MISEV: Minimal information for studies of extracellular vesicles (published in 2018)
MLI: Mean linear intercept
MS: Mass spectrometry
MSCs: Mesenchymal stem cells
NANOG: Type of homeobox transcription factor
Nfe2l2: Nuclear factor erythroid 2-related factor 2
NF-κB: Nuclear factor kappa-light-chain-enhancer of activated B cells
NOTCH: Neurogenic locus notch homolog proteins
NTA: Nanoparticle tracking analysis
OCT: Optimal cutting temperature compound
OCT4: Octamer-binding transcription factor 4
PBS: Phosphate buffered saline
PE: Phycoerythrin
PI3: Phosphoinositide 3-kinase
PDGF: Platelet derived growth factor
Prdx5: Peroxiredoxin 5
qRT-PCR: Real-time polymerase chain reaction
RIPA: Radioimmunoprecipitation assay
SEC: Size-exclusion chromatography
SERPINA1: Serpin protein A1: alpha-1-antitrypsin
SERPINA3: Serpin protein A3: alpha-1-antichymotrypsin
SOX: Sex determining region Y-box
T1alpha: Lung type I cell differentiation gene, also known as podoplanin
TEM: Transmission electron microscopy
TGF-β: Transforming growth factor-beta
TGFBI: Transforming growth factor-beta induced protein
TRA-1–60: T cell receptor alpha locus, also known as podocalyxin
TTF1: Thyroid transcription factor 1, also known as NK2 homeobox 1
UF: Ultrafiltration
VEGF: Vascular endothelial growth factor
VEGFR: Vascular endothelial growth factor receptor
