# Supplementary material for: Effectiveness of extracellular vesicles derived from hiPSCs in repairing hyperoxia-induced injury in a fetal murine lung explant model
Source: Stem Cell Res Ther. 2024 Mar 14;15:80. doi: 10.1186/s13287-024-03687-3 (PMC10941466; doi:10.1186/s13287-024-03687-3)
Supplement: Supplementary file 1 — Additional file 1: Figure 1 hiPSC characterization during the differentiation process, using flow cytometry. Prior to their differentiation, hiPSCs had over 95% positive expression for the pluripotency markers OCT4, SOX2, NANOG and TRA-1-60. At the Definitive Endoderm DE stage (day 3 of differentiation), cells expressed more than 80% positivity for CXCR4 and EPCAM. At the Anterior Foregut Endoderm AFE stage (day 5 of differentiation), 89% positivity for SOX17 and 73% positivity for FOXA2 were obtained. FOXA2 is a key marker of AFE while SOX17 marks the transition from DE to AFE. On day 25, cells had reached the distal lung phenotype stage (diPSCs). Their positivity for pluripotent markers has much decreased. Over 90% positivity for TTF1 and T1alpha, 33% positivity for AQP5 and 4% positivity for SP-C were obtained at this stage. Figure 2 A. Schematic representation of the fetal lung explant model (Abbreviations: BM: basement membrane; FGF: fibroblast growth factor). B. Preserved viability of the explant model for up to 7 days of in vitro culture. Calcein and Ethidium Bromide were used for Live/Dead staining. Imaging was carried out using a Zeiss Observer inverted microscope. Over 85% of viability was maintained throughout the in vitro explant culture, validating its suitability for experimental use during this time period. To note that the study experiment was carried out over the first 3 days of culture. Figure 3 Prdx5, Nfe2l2 and VEGFa gene expression level relative to the internal control GAPDH (delta Cq), based on qRT-PCR. Refer to fold-change relative to the internal control and to the normoxia tissue in Fig. 6. The lower delta Cq is, the higher is the fold-gene expression. Figure 4 Relative normalized expression of IL6, NF-κB1 and TGF-b1, based on qRT-PCR results. These genes are known to be upregulated following hyperoxia injury in vivo, but no changes were observed in our in vitro model. Figure 5 NTA of a PBS sample run through the chromatography column. This [file 13287_2024_3687_MOESM1_ESM.docx]

**Supplemental Figure 1**

**hiPSC characterization during the differentiation process**


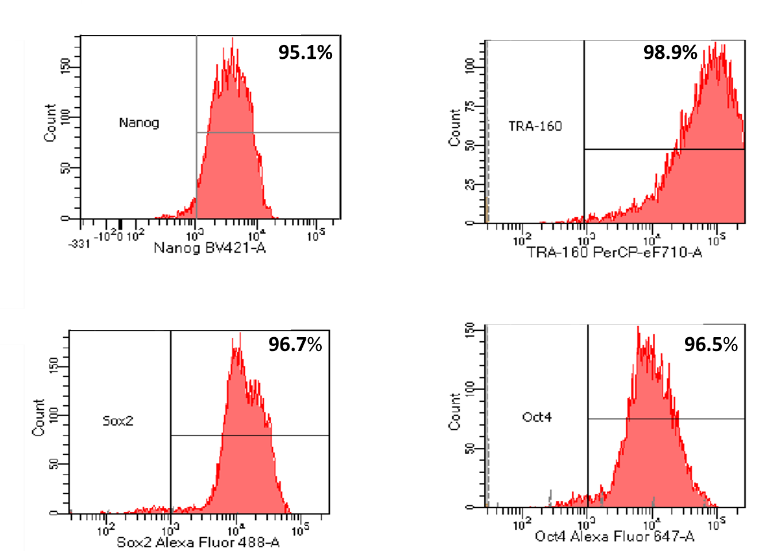

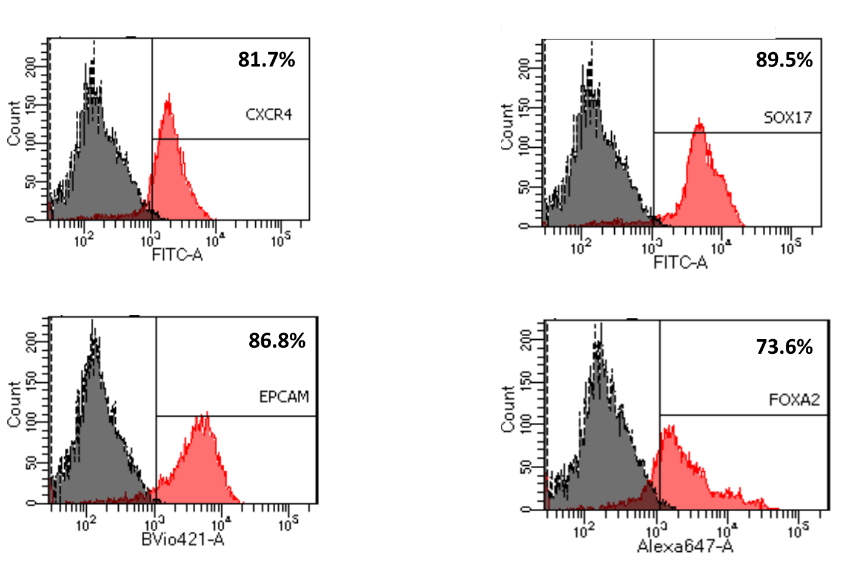

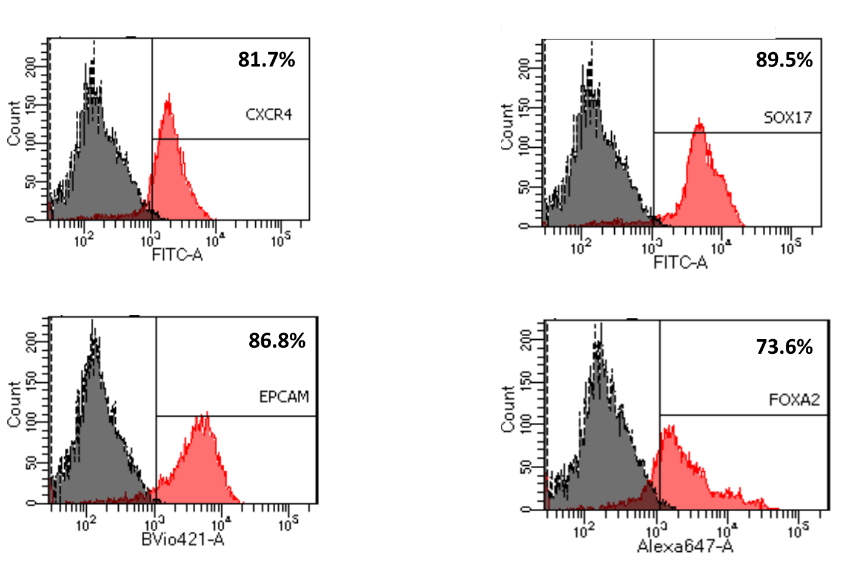

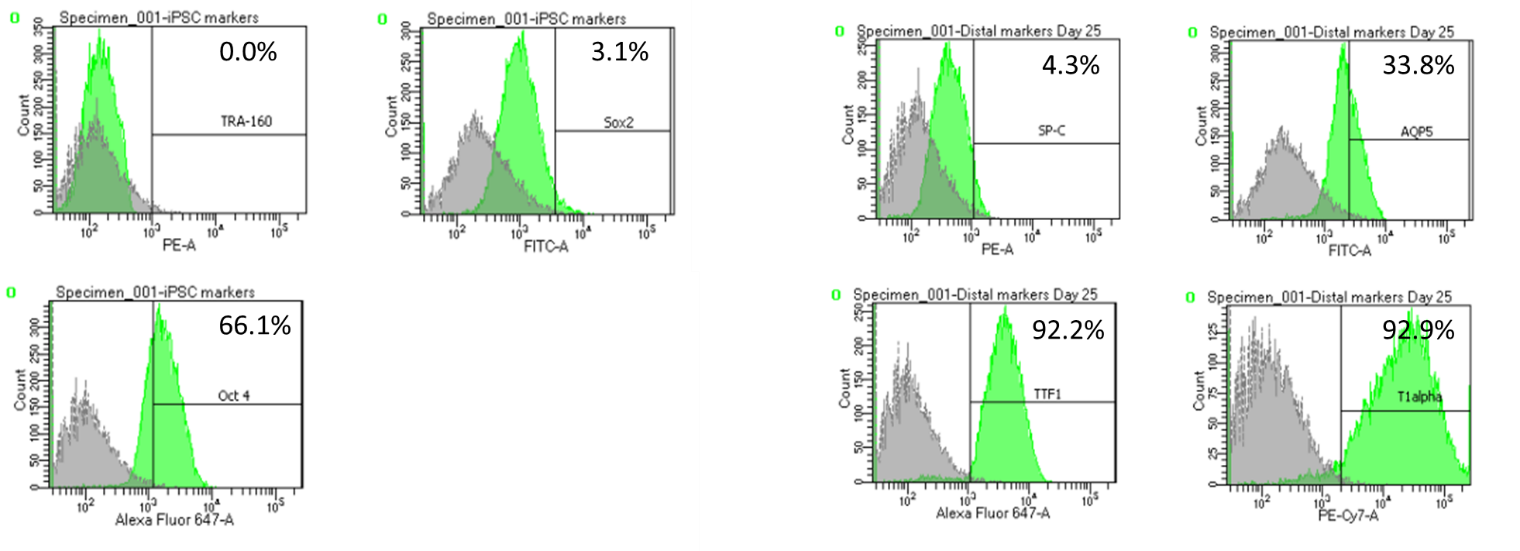


**Day 1 (undifferentiated hiPSCs)**

**Day 3 (DE)**

**Day 5 (AFE)**

**Day 25 (diPSCs)**

**Supplemental Figure 2**

***In vitro* culture of the explant lung model**

**A.**


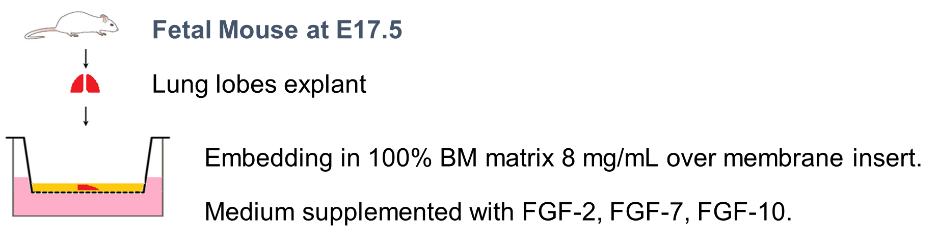


**B.**

**
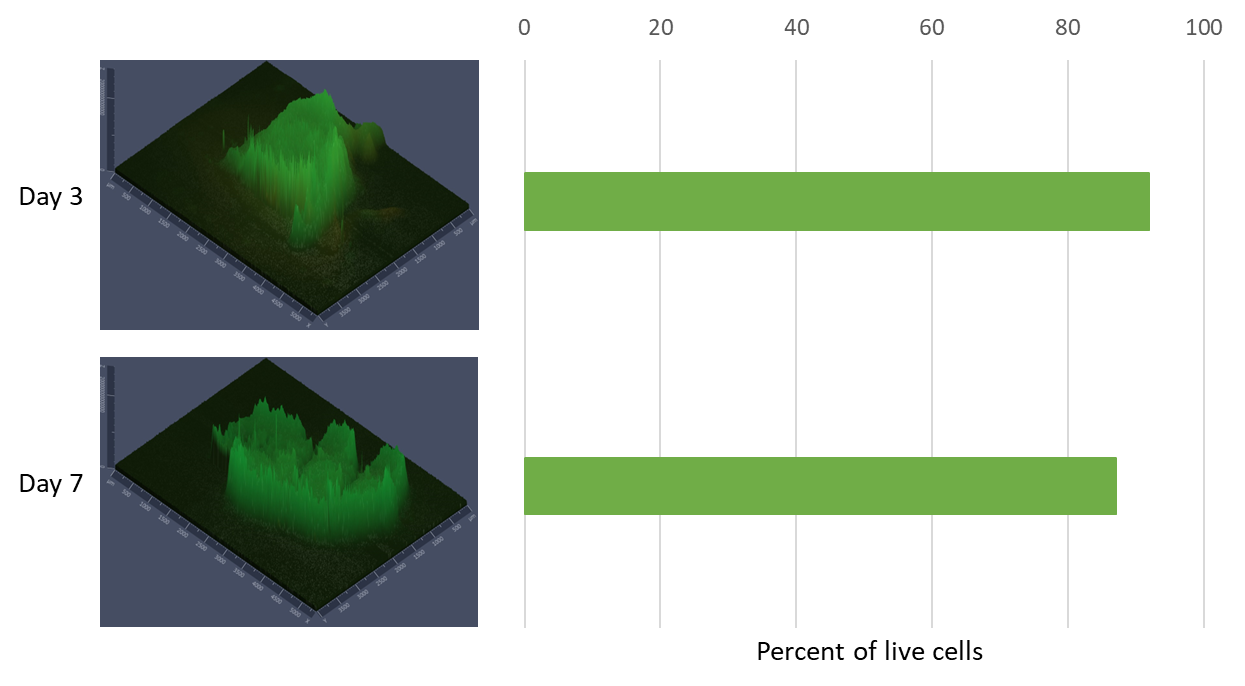
**

**Supplemental Figure 3**

**Gene expression level relative to the internal control GAPDH (Delta Cq)**

**Supplemental Figure 4**

***Relative normalized expression of IL6, NF-κB1, and TGF-b1***

**Supplemental Figure 5**

**NTA of a PBS sample run through the chromatography column**

Concentration: 2.5E+7 particles/mL
Median diameter: 151.3 nm


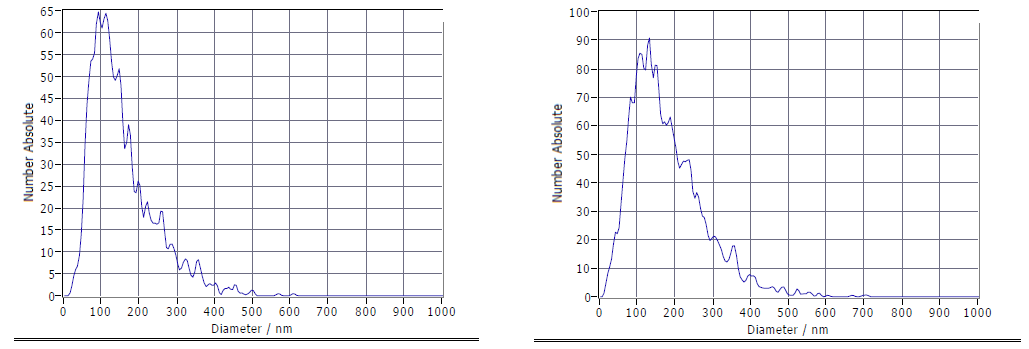

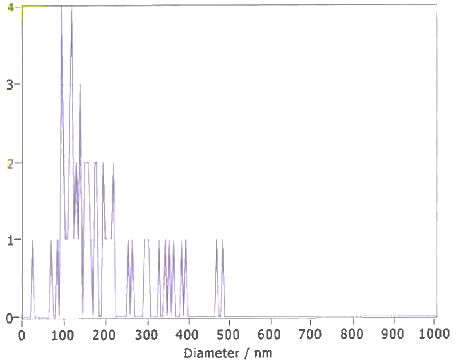


**Supplemental Figures captions**

**Supplemental Figure 1.** **hiPSC characterization during the differentiation process, using flow cytometry.** Prior to their differentiation, hiPSCs had over 95% positive expression for the pluripotency markers OCT4, SOX2, NANOG and TRA-1-60. At the Definitive Endoderm DE stage (day 3 of differentiation), cells expressed more than 80% positivity for CXCR4 and EPCAM. At the Anterior Foregut Endoderm AFE stage (day 5 of differentiation), 89% positivity for SOX17 and 73% positivity for FOXA2 were obtained. FOXA2 is a key marker of AFE while SOX17 marks the transition from DE to AFE. On day 25, cells had reached the distal lung phenotype stage (diPSCs). Their positivity for pluripotent markers has much decreased. Over 90% positivity for TTF1 and T1alpha, 33% positivity for AQP5 and 4% positivity for SP-C were obtained at this stage.

**Supplemental Figure 2.** **A.** **Schematic representation of the fetal lung explant model** (Abbreviations: BM: basement membrane; FGF: fibroblast growth factor). **B.** **Preserved viability of the explant model for up to 7 days of *in vitro* culture.** Calcein and Ethidium Bromide were used for Live/Dead staining. Imaging was carried out using a Zeiss Observer inverted microscope. Over 85% of viability was maintained throughout the *in vitro* explant culture, validating its suitability for experimental use during this time period. To note that the study experiment was carried out over the first 3 days of culture.

**Supplemental Figure 3.** ***Prdx5*, *Nfe2l2* and *VEGFa* gene expression level relative to the internal control GAPDH (delta Cq), based on qRT-PCR.** Refer to fold-change relative to the internal control and to the normoxia tissue in *Figure 6*. The lower delta Cq is, the higher is the fold-gene expression.

**Supplemental Figure 4.** **Relative normalized expression of *IL6*, *NF-κB1* and *TGF-b1*, based on qRT-PCR results.** These genes are known to be upregulated following hyperoxia injury *in vivo*, but no changes were observed in our *in vitro* model.

**Supplemental Figure 5.** **NTA of a PBS sample run through the chromatography column.** This is a negative control sample. Nanoparticles were nonetheless detected and are likely to represent contaminants inherent to the column itself. These particles were much less abundant than those detected by NTA in EV samples (they represent only 2.5% of the total particles detected in EVs) and had more heterogeneous diameters (widespread distribution with large standard deviation). While these contaminants did not seem to have a harmful effect on our lung explant model, their safety remains a concern for clinical translatability and will need to be further investigated.
